# The importance of cultural understanding and practical solutions during the handover of a psychotherapy and psychotraumatology program in Northern Iraq into local hands

**DOI:** 10.3389/fpsyt.2024.1434670

**Published:** 2024-07-25

**Authors:** Gabriel Kornwachs, Martin Hautzinger, Jan Kizilhan

**Affiliations:** ^1^ Department of Clinical Psychology and Psychotherapy, University of Tübingen, Tübingen, Germany; ^2^ Institute for Transcultural Health Science, DHBW, Stuttgart, Germany

**Keywords:** cognitive behavior therapy, PTSD, internally displaced persons, refugee, trauma, psychological, program sustainability, training program

## Abstract

In post war regions, especially in low-income countries, the health care systems often require immediate support. For example, after the terror of the so-called Islamic State of Iraq and Syria (ISIS) in 2014, many internally displaced persons took refuge in the Kurdistan Region of Iraq (KRI). Those displaced by war have had to face the reality that psychotherapy did not exist as a service in the Kurdish health system. Many projects and Non-Government-Organizations (NGOs) that work in post-conflict regions focus on short term and quick response and/or basic psychological services. The implementation of the “Institute for Psychotherapy and Psychotraumatology” (IPP) at the University of Dohuk, follows a long-term approach. The 3-year-program teaches students to become professional psychotherapists, with respect to evidence-based and culturally adapted methods of psychotherapy. To achieve sustainability, the project is working towards handing over the teaching and organizational responsibilities into local hands. This article highlights the chances and challenges during this transition, as well as the importance of cultural understanding and realistic, practical solutions. An honest reflection on existing cultural challenges, e.g. inflexible hierarchical structures or an “old-fashioned” religious view of homosexuality, can then lead to practical solutions. These include winning over local authorities by including them in the process, culturally adapting to customs with the help of educated locals, demonstrating non-authoritarian forms of leadership, and explicitly promoting newly graduated young lecturers into positions of authority.

## 1 Introduction

The *Institute for Psychotherapy and Psychotraumatology* (IPP) was founded in 2016, guided by the idea of introducing sustainable structures for psychotherapeutic treatment into the Kurdish autonomous region of Iraq, a region that provides shelter to around one million internally displayed people and refugees (1), many of whom fled during the brutal attacks of the so-called Islamic State in August of 2014.

### 1.1 Historical Context

The KRI had suffered multiple human rights violations for a long time. The population is burdened. Already during the Saddam regime era, there was a poison gas attack in 1988 that killed an estimate of 5,000 people (2). According to Human Right, 182,000 Kurds were murdered and hundreds of thousands were arrested and deported (3, 4).

Since 2016 three cohort (65 students) have successfully graduated the three-year lasting Master Studies as of today (for a description see 5). A fourth cohort is expected to finish in 2026.

### 1.2 Sustainability

Long term psychotherapy is desperately needed in this region, as the consequences of war and displacement are manifold and long term, such as transgenerational traumatization (6). Some beneficiaries will need long term therapy and thus sustainable resources and providers. Transferring the educational and organizational responsibilities into local hands creates an environment, in which providers and beneficiaries share the same culture, speak the same language, and possibly even share certain experiences. This creates a profound understanding of what is needed, which obstacles may arise and how to address the latter.

To achieve sustainability and the continuation of the project without the initial doners and organizers, the project should one day be run autonomously, without support from Germany, and thus “stand on its own two feet”. A “train-the-trainer”-approach has been initiated with selected graduates from the first cohort. These graduates are being trained to become lecturers, practical and scientific supervisors, and the project’s leaders and organizers. This is an ongoing process, requiring multiple steps, however, many responsibilities have already been taken over by the local staff with continuous support in the background by the initiators. To the authors’ best knowledge, there is no comparable program worldwide.

As the process of handing over the responsibilities into local hands faces several challenges, this article highlights the chances and limitations during that process. It describes the transition process as well as the cultural and political challenges, faced by both students and local lecturers (LL). This article depicts possible solutions, that become visible once those challenges are identified and faced in a culturally sensitive, but also realistic, practical and honest manner. Hierarchical structures, unreasonable political decisions and the consequences of decades of dictatorship are amongst these challenges. It is the authors’ firm understanding that the process of handing over responsibility into local hands, via a careful, thorough, and practical transition, is the key aspect to guaranteeing sustainability and the long-term success of the project (as the initial funding from Germany will eventually end).

As the IPP’s existence can only prevail in the long-term if the local staff take over the full responsibility, a complete withdrawal of the German donors and initiators of the project should happen slowly in a steady step-by-step process and requires the cooperation and goodwill of the University of Dohuk, the Ministry of Higher Education, the Ministry of Health and the Regional Government, political decision makers and local NGOs.

### 1.3 Cultural sensitivity

The *Institute for Psychotherapy and Psychotraumatology* (IPP) aims to provide basic principles, advanced methods, and practical applications of psychotherapy and psychotraumatology. It is broadly known that psychotherapy is sensitive to cultural factors and thus can only be implemented in a society, if the cultural identity, rules of the community, and cultural norms are considered (see 7). Beneficiaries from different cultures, for example from the Middle East with an Islamic cultural background, have different perception of illness and healing. In the Islamic, God-centric view of healing, direct approaches are often via recitation and obeying of the Qur’an, or indirectly via human agents such as Imams, family members, or respected members of the community (8).

The intention to implement the idea, knowledge, and structure of psychotherapy into the KRI can thus only work, if the above principles of cultural sensitivity are followed. For example, the IPP applies these principles in the selection of our students and staff members (mainly former graduates) according to the regional cultural and religious diversity of the society living in and around Dohuk, especially including members of minority groups, such as Yazidi, reflecting the enormous burden they carry due to the experienced various genocides (9). This is crucial, as the selection of the local staff forms the future and should build the future steering-board of the IPP. Additionally, a culturally diverse selection of students and staff indicates that all members of society are welcome as beneficiaries of therapy and will be able to find a suitable therapist who represents their cultural norms, language and/or religion from the pool of therapists trained at the IPP.

### 1.4 The program

The program is guided by the following principles, that (a) the training should be culture and trauma sensitive, (b) a core group of local professionals should be trained so that they can provide training for further local therapists, (c) research should be encouraged to provide more information on needs and project outcomes and (d) that the project should be well coordinated with local leaders, the local government, health care services, and academic institutions (5). Following these principles, the program provides a combination of lectures and practical exercises of specific methods and techniques in Psychotherapy. One key focus of the course is the simultaneous acquisition of both the theoretical background (600 hours) and the practical experience within the clinical field (with 1800 required practical hours in camps, psychiatric hospitals or private practices). During the master program, students learn to provide psychotherapy to beneficiaries under regular individual and group supervision. Furthermore, they undergo an in-depth self-experience process to be mentally prepared and have the necessary resiliency for this challenging profession. Overall, they have to undergo 4200 training units of practical and theoretical learning.

## 2 The chances

Since 2016, the IPP has played an important role in introducing the concept of scientifically proven psychotherapy into Kurdish society in Northern Iraq (Province of Dohuk). An outpatient-clinic has been founded that operates in Dohuk City, is run by seven graduated students, and collaborates on a daily basis with the IPP. At this clinic alone, psychotherapists provide psychotherapeutic sessions to teenagers and adults with a broad spectrum of all psychological disorders (approx. 170 sessions per month). The graduates from three cohorts of the IPP working throughout Iraq have helped to create awareness that psychotherapy can help people that suffer from severe mental conditions, and also contribute to the prevention of further outbursts of violence. It could be argued that this has contributed to de-stigmatizing of mental disorders in this region (5). Graduates of the IPP work in many different areas, holding leading positions as supervising clinical psychologists and psychotherapists in many regionally and internationally operating NGOs throughout the country. Psychotherapy as a concept and a way to alleviate mental suffering is spreading in the region and beyond, and a network of psychotherapists has been established. Still, there are important steps to go, to further guarantee the continuation of the program.

*“The IPP has impacted my personal work tremendously, as after having graduated, I have been working in a high position with a big responsibility ever since. I have worked with hundreds of cases, especially survivors of ISIS captivity and cases that have been severely impacted by war, and have suffered from sexual, physical and emotional abuse. Since graduation, I have been supervising students in camps and teaching them a lot of psychotherapy techniques.”*

(Nouri Saeed Khudur, 33, Psychotherapist in *Springs of Hope Foundation*, Supervisor for IPP Master students in Essian camp and Mam Rashan camp).

*“After becoming a graduate from the IPP and becoming a psychotherapist, I was able to give something back to my community by having helped many members of my community with Post Traumatic Stress Disorder, depression, Obsessive Compulsive Disorder, Generalized Anxiety Disorder and many other severe disorders.”*

(Mayan Ismael Hussain, 34, local lecturer and supervisor at the IPP, has worked for several NGOs, e.g. *Jiyan Foundation*, *Free Yezidi Foundation* and *Step In foundation*).

For a closer look upon the impact that the IPP graduates have on the community please see an article published in the New York Times Magazine article (10).

### 2.1 Why a “train the trainer” concept?

Advocates for sustainable development policy often cite and follow the principle of the American philosopher and pedagogue John Dewey (see p. 350; 11):

*“The best kind of help to others, whenever possible, is indirect, and consists in such modifications of the conditions of life, of the general level of subsistence, as enables them independently to help themselves.”*

Thus, a sustainable policy is often described as helping beneficiaries to enable themselves to be able to help or care for themselves (e.g. 12). Following this principle, beneficiaries will not stay dependent on the initial helpers/donors, and thus real sustainability can be best established. It is the goal of this project, that the German support can eventually withdraw and the project be run by our trained local lecturers. Therefore, the project should be integrated into the framework of a local university system, e.g. the University of Dohuk, which in the long-term would become responsible for both the administration and funding of the institute.

Furthermore, it is well known from decades of studies of psychotherapeutic outcome that the patients’ outcome is rather predicted by factors of therapeutic relationship, empathy, warmth, and alliance than specialized treatment interventions. (e.g. see 13). Given the importance of the interpersonal level it therefore seems crucial that those that best understand the clients’ language, background, customs and culture should also be the ones to provide not only the service of psychotherapy itself but also the teaching of its principles. This includes holding lectures and practicing with the students. Therefore, locals can most effectively bring psychotherapy methods to life by creating their own practical ideas, working methods, and techniques, that build on the ideas they once learned (as former graduates). The LL are best able to adapt well established principles of psychotherapy into their cultural framework. They can create own practical applications of basic therapy techniques and decide how those can be best translated, operationalized, culturally adapted, and filled with life within the language they share with the students and the beneficiaries. The students themselves can then individually and cultural-sensitively apply those techniques on the beneficiaries. Thus, important factors for the success of psychotherapy, such as understanding and building a trustworthy therapeutic relationship, can be both, applied and learned, first between lecturer and student and then between student and beneficiary.

### 2.2 The process of handing over the responsibilities

**Figure 1:**
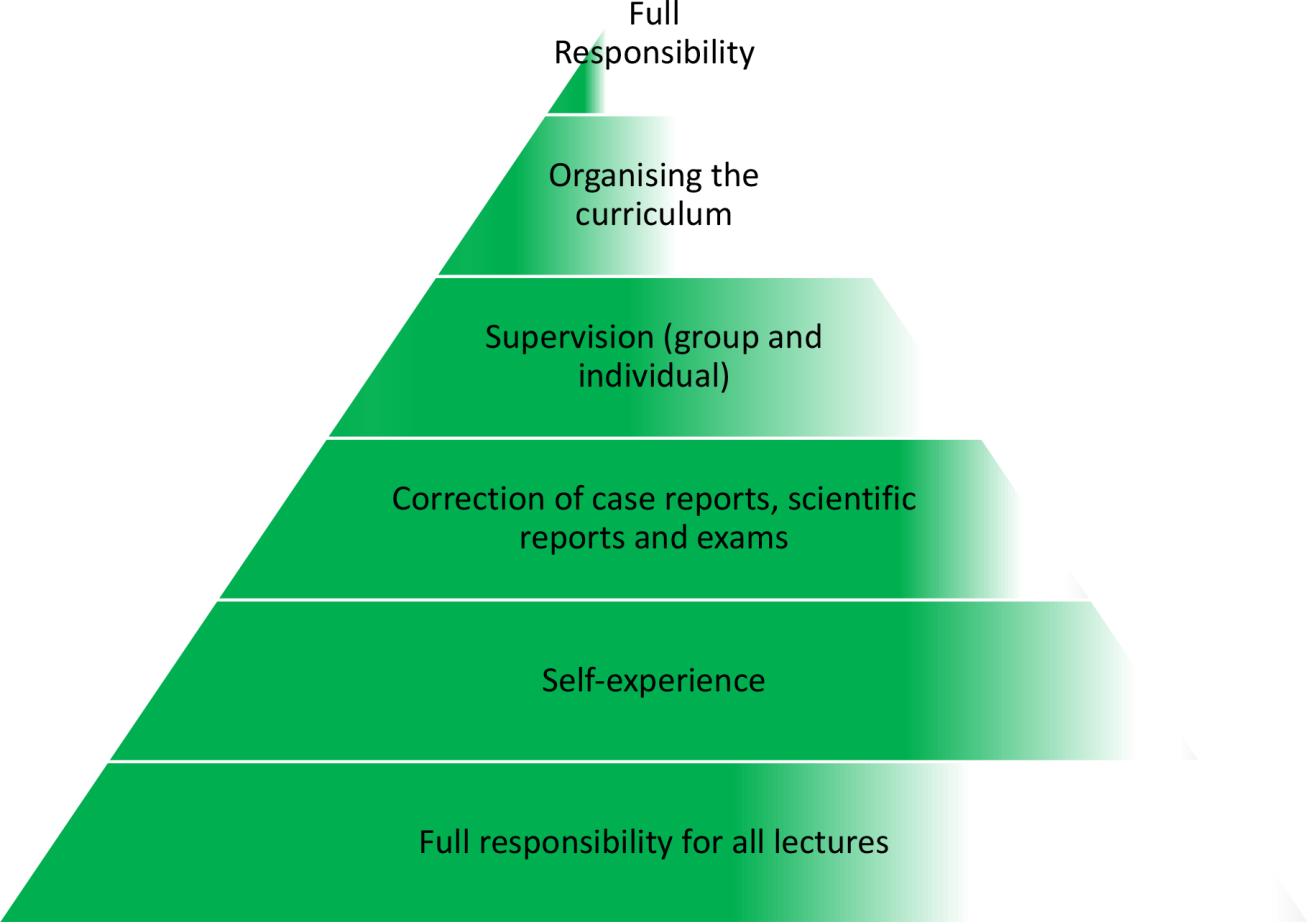
The multiple necessary steps for taking over the responsibility of the project by local lectures. Green color indicates the estimated degrees of progress.

Over the last three years eight selected graduates from the first cohorts have been taught by the authors and other international lecturers to run the program. They have been guided towards taking over the lecturers, providing supervision, running the organization of the curriculum, and in assisting the students in writing case and scientific reports, the master thesis and other exams. Over this period of time, recurrent supervision meetings, including exercises in lecturing, didactics, providing supervision and correcting student assignments, were run. Applying this stepwise process, they were able to take over more and more core-parts of the project (**Figure 1**).

They started off by supporting the external experts as co-lecturers. Under supervision, they then started preparing and holding parts of the lectures. Currently, the majority of most lectures are planned, organized and run by the LL with only minor support from the authors and other international experts in the background.

Initially the LL assisted the authors in correcting exams. In a guided process, they then established their own criteria for the correction of exams and student assignments. Currently, they take responsibility in correcting students’ academic works, with internal back-up-checking within their group and feedback from the German team.

Whilst sharing their experience and receiving feedback from the authors, they provided their first group and single supervision. Currently, supervision is run by both the local team and other international experts.

### 2.3 The adaptation of the curriculum

Today, most topics are covered by the LL with international experts visiting the project from time to time. Consequently, the selection of the lecturers has moved from a mainly international dominated field to a more and more local one, respecting the local variety of ethnicities, cultural norms and language. Today, the selection of lecturers comprises of a multidisciplinary team, with mainly local experts. The mental health sector in this region is still very much dominated by physicians and traditional healers. Including local psychiatrists in the curriculum, has helped not only to raise their acceptance towards this “new” approach, but also to advocate for psychotherapy as a useful approach. This has indirectly helped the reputation of psychotherapy as a profession in this region. As a consequence, over the last years, the collaboration and acceptance within the local psychiatrists and religious authorities has significantly improved, with not only psychiatrists but even traditional healers (sheikhs) referring clients to our graduates.

Thus, the selection of lectures and topics of the curriculum (a) represents and respects the cultural norms and ethnic variety, (b) includes a variety of disciplines, but also (c) has to adapt to regional customs, hierarchical structures, and every-day life circumstances of working in this field.

## 3 The challenges for the students

### 3.1 Pending legal recognition as a new profession in KRI

Although the recognition and reputation of psychotherapy as a profession in this region has rapidly increased in the last years, and overall satisfaction with received mental health care is high (14), still the legal recognition and the legal framework for psychotherapy as a profession is pending. It remains unclear what the legal rights and responsibilities for working psychotherapists are. There are no national guidelines, which leaves the therapist working within unclear conditions. Consequently, they have no choice but to rely on principles which they have once learned during psychotherapy courses, which are western standards. For example, it remains legally unsolved if psychotherapists are allowed to make a diagnosis. Thus, it is common practice that most well-trained psychotherapists still work under supervision of a psychiatrist.

Especially frustrating for students is the fact that the University procedure to hand out official certificates of graduation is slow. It seems as if bureaucratic barriers are hindering the graduated psychotherapists from working under clear legal circumstances because they simply have no certificate yet. Although their work is highly appreciated by many NGOs and by the beneficiaries themselves (14), psychotherapists often work under the precarious working condition of not having any description of their rights and duties for their work, besides other problems, such as not having a designated room (safe space) for psychotherapy inside the camps.

Additionally, until today there are no rebates for psychotherapy as no health insurance exists in the health care system in this region. Thus, psychotherapy as a service is either fully paid by international donation or by the individual beneficiaries themselves.

“Strengthen(ing) sustainable economic activity” is named as one key factor in the German Sustainable Development Strategy (2021). This includes, that only if members of a profession meet acceptable working conditions, and can make a living from the job, the profession will be attractive. Only if psychotherapy is officially recognized (with its official certificates) and represents an honorable profession with the necessary conditions for the beneficiaries and the deliverers, will the profession itself be able to endure. Legal steps are required to improve the situation of psychotherapists in this region. This would mean developing a legal scaffold with clear guidelines for psychotherapists on the one hand and more supporting conditions for beneficiaries on the other hand.

Interim measures include a preliminary certification from the University. Furthermore, the collaborating NGOs are well informed and are well aware about the pending certification and legal recognition. Most NGOs are also aware about the curricula at the IPP and specific courses. They acknowledge the high level of skills the graduates provide and hire them for high positions. This acknowledgement alongside with a strong community spirit, that IPP graduates share provides the local psychotherapists with a sense of professional legitimacy and security in their practice.

### 3.2 Hierarchical structure and resources of authority

The university system in this region is based on highly hierarchical structures. It sometimes appears as if some higher ranked decision makers struggle to implement practical solutions that would thereby create agreeable working conditions, fruitful for scientific research and progress. Instead, they prioritize maintaining given structures, protecting their own positions and therefore sticking to established rules and bureaucratic barriers. It seems challenging for some to regard the university as a free space that allows and acknowledges not only the freedom of research but also the possibility for students to work in this field and gain positions that are representing their valuable work. Nevertheless, the goodwill of certain political figures often remains a key aspect to finding solutions. Against the will of certain hierarchical figures, some ideas seem to be condemned to fail, no matter the initial quality of the concept.

This affiliation to hierarchical structures also runs counter to a modern concept of psychotherapy, according to which the beneficiary meets on an equal footing with the therapist. In contrast to the Western concept of the beneficiary-therapist-relations, in Kurdish culture there is a hierarchical perception of this relationship, due to factors such as age, gender and authority (7). This creates a challenging environment for the advocates of the establishment of modern psychotherapy. In the Kurdish culture, position and age both represent very strong sources of perceived authority, indicating that even if a person is highly competent, if they are lacking a certain age and/or of a hierarchically high position, it makes it very hard for this person to receive trust, respect and authority.

This hierarchical perception of competence biases the students, too. Often the students a-priori expect more expertise from guest lectures from overseas or other authorities, such as local psychiatrists, than from the mostly younger LL. The mere fact that most of the LL are not much older than the students, is the underlying reason as to why the LL having to strive especially hard to gain authority. To face this tricky condition, again it seems important that respected figures (the “international experts”) display in an obvious, visible and clear manner that they trust in the competence and expertise of the local staff. To name one concrete example: The international expert could be present during the lecture, mainly supporting and sitting in the background, and then in specific moments display and emphasize the importance of what the lecturer has just said, e.g. by verbal or non-verbal validation, such as murmuring agreement, nodding the head, etc.

Therefore, both winning over the acceptance and the goodwill of certain protagonists and fostering the perceived authority and respect in our LL are necessary steps. It is crucial to meet those protagonists’ need for control (e.g. by including them into organizational matters), whilst not allowing the regional university to micromanage the project bureaucratically and organizationally. Additionally, providing the LL with all necessary legitimation (“proof of power”) seems crucial. This involves (a) profoundly and visibly promoting the rather young lecturers with the necessary authority explicitly in front of the students, and (b) fighting for their rights within the University structure.

### 3.3 Difficulty to “embrace freedom” and having an own opinion

In the beginning, many students often strived with questions that are considered as “open” or transfer-questions, when critical thinking is more important than only “digesting” knowledge and learning by heart. Years of dictatorship and suppression of the expression of free speech as well as the above described still existing rigid system of hierarchical authority seems to have impacted the understanding of knowledge processing, in a way that presented contents are often regarded as given facts. In the latter understanding, those facts should be digested and not challenged with one’s own critical perspective.

This perception and understanding may be overcome by (a) presenting in a confident way (meeting the wish for a strong and thereby accepted lecturer) and at the same time (b) repetitively encouraging the students to bring in their own thoughts and ideas, to think critically and even to be allowed to have a different opinion than the lecturer (the person of authority). Thereby, the lecturer may represent a role-model, by not avoiding critical questions, and openly reflecting on difficult matters. Admitting that certain matters/issues may have to stay unsolved, can then even be perceived as a sign for strength instead of weakness.

### 3.4 Perception of unfairness

Students often seem to have a strong and sometimes overwhelming perception of being treated unfairly compared to their peers. Sometimes they even perceive this as discrimination against their own person, their cultural or religious minority or simply as an unfair judgement of their skills and competencies by the corrector. As a consequence, the competence of the local lecturer to evaluate the exams or assignments is often questioned or even directly attacked.

Justice has been described as an enormously important factor in the psychotherapeutic treatment of traumatized people after war and crisis (15). Justice seems also crucial towards the helpers (students), especially when implementing such a program with students form multiple ethnic backgrounds. Therefore, it is the authors’ experience, that the correctors of exams should (a) be explicitly transparent about the process of evaluation, e.g. explain the criteria, (b) agree with a double check and therefore represent a corrective experience towards patriarchal structures, and (c) stand by the assessment if after a fair re-evaluation the questioned decision has been proved correct (or corrects the assessment if an error has occurred). Although this may sound obvious, it may represent a new and therefore highly valuable experience for our students. Thus, the correction needs to balance out a transparent and straight-forward leadership on the one hand with an empathetic understanding of the students’ concerns on the other. The latter can be difficult as it needs to address both the hierarchical structures within which the students grew up in, and the students’ feeling of being treated unfairly and the accompanying culture of verbalizing this feeling by complaining and contesting.

### 3.5 Torn between religious traditions and modern ethics

Local students as well as local lecturers/supervisors face the challenge that certain cultural perceptions seem to be hard to integrate into the ethics and moral standards of (western) psychotherapy. For example, homosexuality is often seen as a disorder, as pathological deviation that should be cured by someone. This is visible in discussions or supervision sessions with our students. Sometimes our psychotherapy students receive referrals from psychiatrists that send their patients to the psychotherapist with the mandate that they should “solve the matter”, i.e. cure the beneficiary from the illness of homosexuality. Students are then torn between the culturally deeply ingrained understanding that homosexually is a disorder and the perspective of modern psychotherapists (we cannot and should not treat the sexual preference of a person, if no one is harmed by the latter). In such cases, the treatment planning can be complicated as (a) the beneficiary and the therapist are both culturally rooted and shaped by his/her experience, and (b) the possible options for a beneficiary within this culture are certainly different than in most western societies (e.g. an outing could be acutely dangerous).

Again, open discussions and reflections are necessary to help the therapist to deal with the case and the beneficiary’s dilemma. Still, such reflective discussions and supervision sessions are only possible if the lecturer (as a role model also for a modern psychotherapist) is not judging and not taking the position of an “almighty, wise healer” that knows everything best. Western principles cannot overrule the daily customs and realities of every-day life (supervision needs to consider this). Only then, in our experience have open discussions and supervision been shown to work in this traditional culture when dealing with those delicate topics. In such reflections or supervision sessions, the students then felt comfortable to openly share their concerns. They can then profit on a personal level and the therapy process can proceed via discussing a realistic treatment plan (e.g. 16).

On the one hand, cultural values have to be respected and integrated. On the other hand, educators and supervisors have to ensure, that professional and ethical standards are met. Therefore, a safe space should be created that enables that those conflicts can be transparently discussed within the supervision, but also within group discussions and lectures. Especially when these standards conflict with local beliefs and practices, psychotherapists have to ensure that certain standards as confidentiality, limited self-disclosure, and holding personal distance towards the beneficiary are kept.

## 4 The challenges for the local staff

Until today the project suffers from political constraints, including bureaucratic limitations such as the condition that only graduates with a Doctorate are allowed to teach Master Students. This is a very challenging requirement as the options to participate in research projects locally, in order to complete a PhD, are limited. Consequently, the LL have to gain a doctoral degree in order to one day officially receive the permission to run the project. To complete a PhD, candidates have to spend two full years abroad during their research. By doing so, in the meantime they would not be able to stay as lecturers and psychotherapists in Iraq, where the demand requires every single therapist.

Until now, experienced psychotherapists can contribute to teaching and supervising, when a supervising expert with the required degree is available in the background. In the long-term, it might be necessary that for a certain time one or two potential future Institute leaders will have to pause their crucial work in Iraq during obtaining the required degree. Consequently, for the future they could then become the necessary experts in the background.

### 4.1 Collaborating with psychiatrists in every-day routine

In the beginning stages, our students and later graduates have faced the issue of local psychiatrists fighting for their positions, patronizing, and undermining the authority of this new profession and its protagonists. With the growing reputation of the project and the valuable work of our students and graduates, this matter has certainly improved. Still, some psychiatrists understand themselves as the only protagonists in the position to diagnose (This is not helped by the fact that up until today, in KRI there is neither legal acknowledgment for psychotherapy as a profession nor any official guideline for rights and duties for psychotherapists in KRI). Including psychiatrists into our project (e.g. as lecturer and exam holder) and collaborating with them within the outpatient clinic has helped to win a broader trust and sympathy towards this new profession. Furthermore, it serves as a role-model of a fruitful collaboration between the professions of psychiatry and psychotherapy for the future, not only academically but also in the out- and in-patient setting in daily practical routine.

### 4.2 Challenges in supervision

During supervision students often appear to feel as though they are being tested. As a consequence, instead of openly discussing the case they try to “shine” and present themselves in a favorable light by explicitly talking about the positive progress rather than the challenges and difficulties. This is probably due to the strong hierarchical structure in academia and the students’ prior experience of being patronized in harsh oral exams. In contrast to the oral exam procedures in western universities, the authors found that the oral exams at the University of Dohuk were often harsher and more oriented around highlighting the deficits than the assets of the students. This can be addressed by repetitively clarifying that supervision is not an exam. In addition, it takes a huge effort from the supervisor to create an error-tolerant environment that is characterized by benevolence (e.g. 16). It also requires that supervisors not only help the student to critically reflect on the therapeutic process and see the realistic limits of psychotherapy, but also see small steps as therapeutic progress. The supervisor needs to acknowledge the beneficiary’s and student’s efforts, and praise the students for achieved goals (even if they are small). Those “small steps” include for example, if beneficiaries show up to therapy sessions, that they display initial steps towards activation (e.g. to participate in a sewing or computer course within the camp), if they are able to talk about options or even simply that their symptoms do not deteriorate within the course of therapy.

Students are trained to be able to reflect on the therapy process itself, the interaction with the patient and the therapy’s influence on their own psyche and well-being. In numerous supervision sessions it has become visible how important it is -besides a profound supervision concerning the case- to support and affirm the students on an emotional level. It is absolutely crucial, besides fostering the student’s therapeutic skills, that the supervisor explicitly and authentically demonstrates respect for the students’ important, tough, and mentally draining work.

Within this context, self-care for the therapist should be directly addressed. Supervision, alongside self-experience, needs to address whether or not the therapist has enough resources (e.g. social contacts, hobbies, sports etc.) to build up enough resiliency. This seems obvious, but its importance cannot be overstated, considering the fact that the therapist will often face grim dilemmas due to the nature of the beneficiaries’ situations (e.g. cases of domestic violence that have no realistic chance for escape due to financial and cultural constraints). This can lead to a sense of helplessness- also for the therapist. Therapists cannot “solve” such situations. Instead, they can “only” provide emotional support by containing and validating the suffering, standing alongside the beneficiary, and thereby helping the beneficiary to withstand the inevitability of the situation (17).

### 4.3 Secondary trauma

Additionally, in many cases our students have had direct or indirect traumatic experiences that come into play during supervision or therapy sessions. Psychotherapy in general, but especially in this region, is an extremely emotionally draining job, which can lead to secondary traumatization (18, 19). Therefore, a profound and thorough process of self-experience is crucial for the students. Throughout the day-to-day routine of being a psychotherapist, self-care and emotional support will remain vitally important.

Secondary traumatization is another argument, why supervision or later intervision should be provided not only as technical but also emotional support. Supportive factors can include spending enough time with loved ones, being active with hobbies and sports, etc. Furthermore, students undergo a profound self-experience training to fully be aware of own strengths and resources, but also own conflicts, triggers, and core-beliefs that might impact their professional work. In a study by Greinacher et al. (20) social support was found to be a key factor in developing resilience against secondary traumatization. Self-care activities and emotional support are often reported by our students and local psychotherapists to be immensely important when dealing with severe cases, especially with PTSD cases on a daily basis (21).

### 4.4 Cultural understanding of the healer, demythologization of the therapist and working mechanism of therapy

Many beneficiaries show reservations regarding their therapist’s gender, religious affiliations, ethnicity and even country of origin (14). Thus, personal attributes of the deliverer of psychotherapy (“healer”) play an enormously important role. Young therapists sometimes underestimate the fact that building up a trustful relationship usually takes time. They often want to help quickly as they might regard themselves as someone who has to come up with a “quick solution”. This urge to help quickly appears to stem from the traditional understanding of healing in the region, which centers around the power of magic, religious rituals, and traditional ceremonies (see 7). Within our project and within our students, this traditional approach meets with an evidence-based understanding of modern psychotherapy. The local therapists have to become experts in both approaches and integrate this fusion into a modern psychotherapy that accepts and adapts to traditional cultural norms.

Those cultural expectations of healing meet the ethical therapeutic boundaries. Students are therefore trained in specific lectures about cultural competences. In those lecture as well as within the group and single supervisions strategies for respectfully challenging harmful cultural norms are discussed (e.g. cultural adequate socratic dialogue). In this regard, it is even more important to teach the supervisee that more often than not, there are no “solutions” to some conflicts and dilemma situations, which may run counter to the idea of a magic healer who always comes up with a full explanation and solution (7). On the one hand, a certain demythologization of the therapist seems therefore unavoidable. On the other hand, this allows other valuable working mechanisms of psychotherapy to take place: Even though there might not be a solution to a problem, a clarification of options can still take place, and small steps towards the goals may be possible and also be regarded as valuable progress. It can already be a very important step if the beneficiary learns that they are not being judged for who they are, what they feel, or what they have experienced. Being able to see such a corrective experience as a success within therapy, is something that supervisors need to explicitly teach their supervisees, as such experiences may seem counterintuitive when compared to the culturally widespread idea of a mighty “healing” figure or “curer”.

## 5 Summary

The article discussed chances and challenges during the process of handing over the IPP into local, Kurdish hands. It depicted changes and limitations in light of the cultural, religious and political challenges, describing practical ideas to overcome certain problems as well as proposing reasonable solutions. The key aspects of the transition from an initially guided Western project into the Kurdish community include (a) the adaptation according to the principle that the teaching of culturally-sensitive methods of psychotherapy best works if administered by locals. The Western principles of psychotherapy can only take root, if locals translate the once implemented basic ideas into practically and culturally functioning methods; true to the motto: seeds best thrive on local soil. On the other hand, the importance of (b) cultural understanding leads to (c) honestly acknowledging the problems instead of naively overseeing cultural challenges. An honest reflection can then lead to (d) practical solutions, such as winning over local authorities; culturally adapting to customs with the help of educated locals; providing a role-model for being a respected but non-authoritarian leader; and explicitly promoting new LL to positions of leadership and authority. It is the only way to guarantee sustainability and the long-term survival of the IPP, if the LL take over full responsibility. Furthermore, if the multiple advantages of LL are taken into account, it is the firm understanding of the authors that this would also be the best possible solution. The World Health Organization states in their report, that one of the main challenging factors to disseminate mental health support programs in Iraq is the lack of continuous financial support (22). As the German funding will eventually end, the sustainable continuation of this project will only be achieved, if (a) the project is fully integrated within the local systems, namely the mental health system and the Ministry of Higher Education (University system), and (b) if it is fully financed by the local government, University, Ministry of Higher Education, and Ministry of Health, including the salaries of the local staff. Although relentless efforts have been made, full financial commitment by the local authorities is still pending.

## Data availability statement

The original contributions presented in the study are included in the article/supplementary material. Further inquiries can be directed to the corresponding author.

## Author contributions

GK: Conceptualization, Data curation, Formal analysis, Investigation, Methodology, Project administration, Resources, Supervision, Validation, Visualization, Writing – original draft, Writing – review & editing. MH: Data curation, Investigation, Methodology, Project administration, Resources, Supervision, Validation, Writing – review & editing, Formal analysis. JK: Formal analysis, Funding acquisition, Investigation, Methodology, Project administration, Resources, Supervision, Validation, Writing – review & editing, Data curation.
